# The effect of bodyweight exercise on 24-h glycemic responses determined by continuous glucose monitoring in healthy inactive adults: a randomized crossover study

**DOI:** 10.1038/s41598-023-48063-y

**Published:** 2023-11-28

**Authors:** Fiona J. Babir, Michael C. Riddell, Larissa M. Adamo, Douglas L. Richards, Martin J. Gibala

**Affiliations:** 1 Department of Kinesiology, McMaster University, Hamilton, ON L8S 4L8 Canada; 2 School of Kinesiology and Health Science, York University, Toronto, ON M3J 1P3 Canada; 3 Department of Medicine, McMaster University, Hamilton, ON L8S 4L8 Canada

**Keywords:** Physiology, Metabolism

## Abstract

Vigorous intermittent exercise can improve indices of glycemia in the 24 h postexercise period in apparently healthy individuals. We examined the effect of a single session of bodyweight exercise (BWE) on glycemic responses using continuous glucose monitoring (CGM) under controlled dietary conditions. Healthy inactive adults (n = 27; 8 males, 19 females; age: 23 ± 3 years) completed 2 virtually supervised trials spaced ~ 1 week apart in a randomized, crossover manner. The trials involved an 11-min BWE protocol that consisted of 5 × 1-min bouts performed at a self-selected pace interspersed with 1-min active recovery periods or a non-exercise sitting control period (CON). Mean heart rate during the BWE protocol was 147 ± 14 beats per min (75% of age-predicted maximum). Mean 24 h glucose after BWE and CON was not different (5.0 ± 0.4 vs 5.0 ± 0.5 mM respectively; p = 0.39). There were also no differences between conditions for measures of glycemic variability or the postprandial glucose responses after ingestion of a 75 g glucose drink or lunch, dinner, and breakfast meals. This study demonstrates the feasibility of conducting a remotely supervised BWE intervention using CGM under free-living conditions. Future studies should investigate the effect of repeated sessions of BWE training or responses in people with impaired glycemic control.

## Introduction

Glycemic control can be defined as the ability to maintain circulating blood glucose concentrations within an optimal range, both in the fasted and post-absorptive states^1,2^. In a clinical setting, glycemic control is typically assessed by measuring fasting blood glucose concentration or hemoglobin A1c levels^2^. The former provides a snapshot of glucose control in an acute fasted state and the latter reflects the average glucose exposure to red blood cells over a 3-month period. Neither measure provides insight into acute glucose changes associated with food intake, stress, or exercise. Continuous glucose monitoring (CGM) is a method that relies on frequent measurements of interstitial glucose concentration to gather comprehensive information regarding both fasted and postprandial glucose responses in a free-living situation^2,3^. The nature of CGM also makes it useful for measuring acute changes in glycemia following interventions involving nutritional manipulation or exercise^3^.

Regular physical activity is an effective strategy to improve glycemic responses^4^. The acute effects of exercise on glucose responses are influenced by numerous factors related to the “dose” including the intensity, duration, and modality^5^. This can be further influenced by the nutritional state of the individual, and in particular, carbohydrate intake before and after the exercise session. Some studies have demonstrated the potential efficacy of relatively brief but vigorous-intensity exercise on acute glucose control measured with CGM. Little et al.^6^ showed that in individuals with overweight or obesity, a single session of intense intermittent cycling exercise (10, 60-s bouts at ~ 90% of peak heart rate (HR)) improved the postprandial glucose responses determined using CGM over the subsequent 24 h (h). Other work has examined the effect of brief bouts of bodyweight exercise (BWE), which may be a more practical method of physical activity since it requires no specialized equipment. Barillas et al.^7^ had healthy, young participants perform a BWE session involving 5 sets of 10 squat jumps and then examined glycemic responses immediately after ingestion of a 75 g oral glucose drink^7^. Capillary blood glucose was reduced by ~ 1.0 mM 15 and 30 min after drink ingestion in the BWE compared to the control sitting condition^7^. Solomon et al*.*^8^ also showed the benefits of performing an acute bout of BWE after breakfast in healthy adults using CGM. In the 2 h following BWE, postprandial mean glucose, area under the curve, and glycemic variability were lower compared to the no exercise control condition^8^. These two studies^7,8^ examined glycemic responses for up to 2 h after exercise however, the potential effect of acute BWE on 24 h glycemic responses is unknown. Vigorous intermittent exercise may acutely increase blood glucose immediately after the session^9^. Hermansen et al*.*^9^ found that venous blood glucose concentration increased by ~ 5.0 mM in young adults after a single exercise session involving 5, 1-min maximal running intervals interspersed with 4-min recovery periods. However, the acute effect of brief, vigorous BWE on meal-related glycemic responses and overall 24 h mean glucose profiles in healthy young adults is unclear and warrants further research.

To our knowledge, no previous study has used CGM to probe the effects of acute BWE on glycemic responses over a 24 h period after the exercise session has been performed. We therefore investigated the potential for a brief BWE protocol to alter acute glucose responses using CGM. The primary outcome was 24 h mean glucose. Secondary outcomes were 2 h postprandial mean glucose, peak postprandial glucose, the maximal meal-related glycemic excursion, and glycemic variability. Glycemic variability was assessed through the mean amplitude of glycemic excursions (MAGE), the 24 h glucose standard deviation (SD), and the coefficient of variation (CV). We hypothesized that mean 24 h glucose would be lower after the BWE intervention compared to a control sitting (CON) condition. We also hypothesized that postprandial glucose means and peaks as well as meal excursions would be lower after BWE compared to the CON condition. Lastly, we hypothesized that glycemic variability would be lower in the BWE condition compared to the CON condition.

## Methods

### Participants

**Figure 1:**
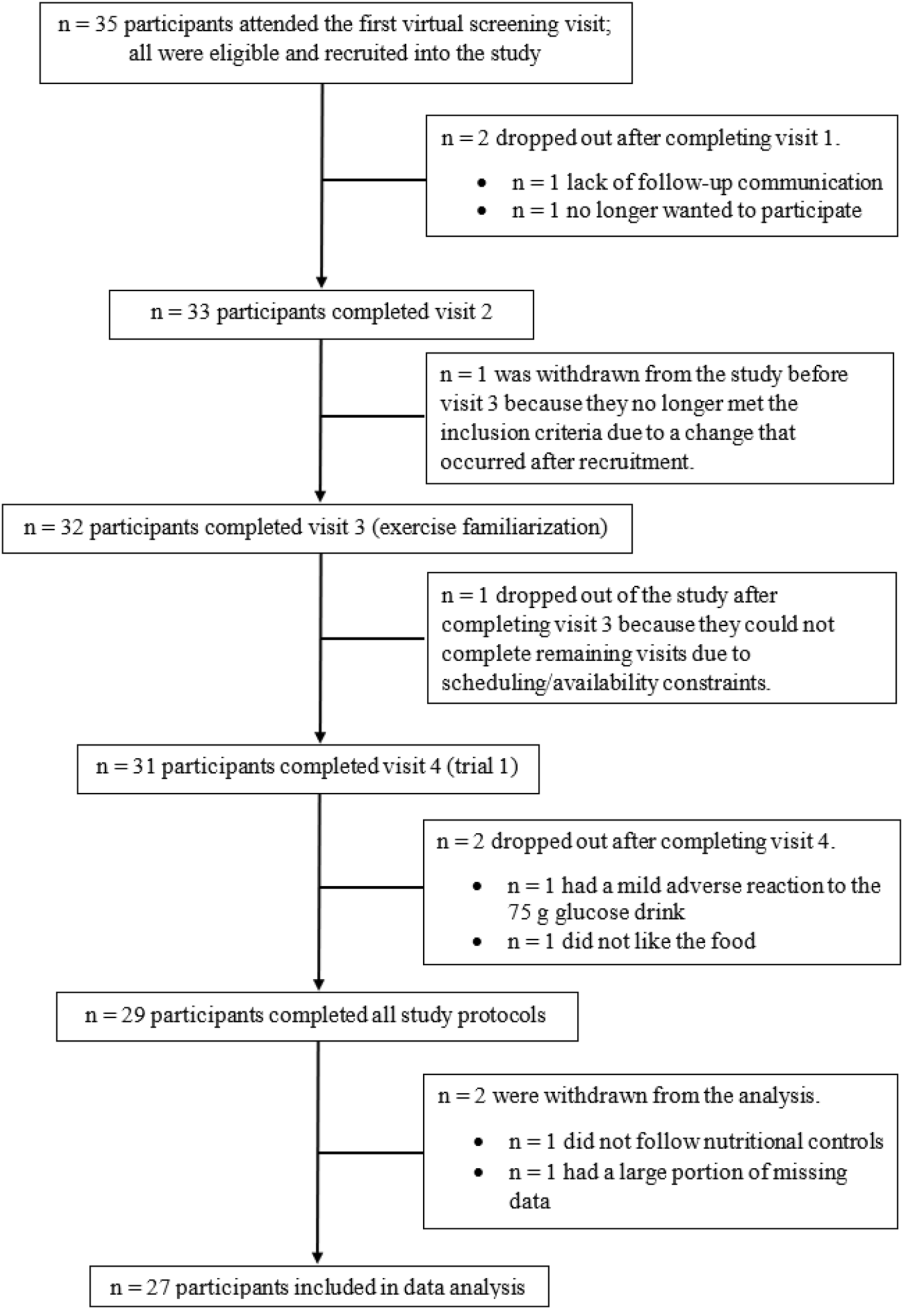
Study CONSORT diagram.

Twenty-seven healthy young adults volunteered to participate after providing signed informed consent. Baseline descriptive characteristics of the participants are shown in Table 1. A calculation performed using an online program (G*Power; version 3.1.9.7) for a one-tailed, dependent means (matched pairs) t-test estimated that a sample size of 27 was required to detect a medium effect size (dz = 0.5) with 80% power at an alpha level of 0.05. A medium effect size was deemed reasonable based on determinations made in G*Power using our hypothesized minimum meaningful difference of 0.2 mM and typical means and SD reported in the literature for 24 h mean glucose. The inclusion criteria were as follows: aged 18–35 years, deemed inactive based on not meeting the aerobic physical activity targets in the Canadian 24-h Movement Guidelines for Adults^10^, and cleared to participate in physical activity per the Canadian Society for Exercise Physiology Get Active Questionnaire^11^. Participants were recruited from the community surrounding McMaster using posters, word of mouth, and social media. A CONSORT diagram summarizing the total enrolled participants and the number included in the final data analysis is shown in Fig. 1. The study was approved by the Hamilton Integrated Research Ethics Board (project # 13864) and this research was performed in accordance with our ethics board guidelines. This study was registered prior to participant recruitment (ClinicalTrials.gov; NCT05144490) on 03/12/2021. Data collection for this study occurred between December 2021 and May 2022.

**Table 1:** Participant descriptive characteristics.

Characteristic	Data
Sex (males/females)	8/19
Age (years)	23 ± 3
Body mass (kg)	70 ± 15
Height (cm)	168 ± 10
Body mass index (kg/m^2^)	25 ± 4

Data presented as means ± SD, except for participant sex.

### Study overview

**Figure 2:**
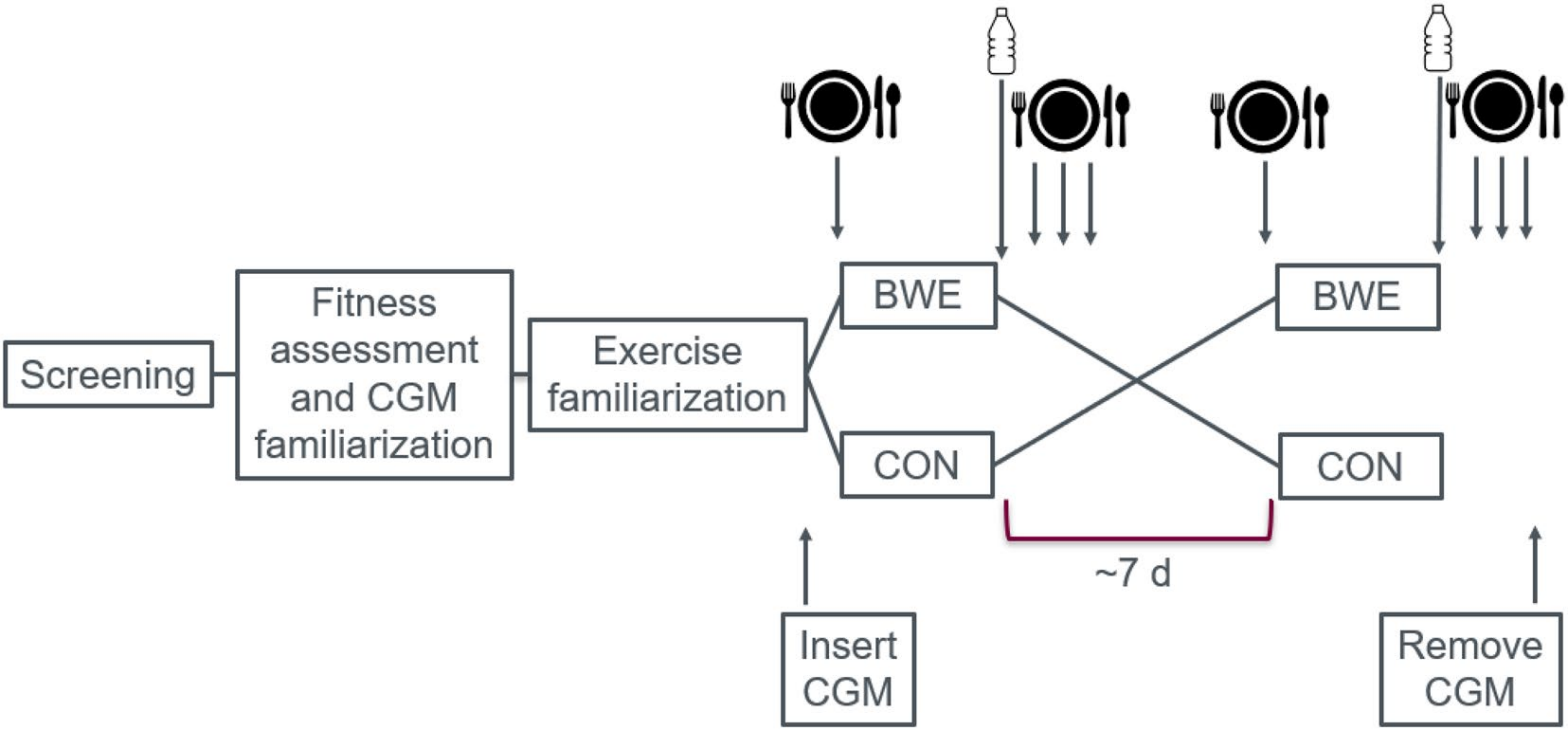
Overview of the acute within-subjects crossover design. Each participant completed a virtually supervised BWE and CON trial with the CGM device inserted to measure glycemic responses in the 24 h following each trial. Plate and cutlery = pre-packaged, individualized, controlled meals. Bottle = 75 g oral glucose drink. *BWE* bodyweight exercise, *CGM* continuous glucose monitoring, *CON* control, sitting.

This study involved a within-subjects, crossover design where each participant completed both a BWE intervention and a non-exercise CON condition in random order. Simple randomization was performed using random number generation and ordering of the two trials within Excel by a researcher in our lab not directly working on this project. The study included 6 phases, each of which involved either a virtual interaction or in-person visit to the Human Performance Laboratory at McMaster University. The phases were: (1) screening (virtual), (2) fitness assessment and CGM familiarization (in-person), (3) exercise familiarization (virtual), (4) first experimental session (virtual), (5) second experimental session (virtual), and (6) CGM removal (in-person). Figure 2 provides an overview of the study design and the various phases. The exercise familiarization was completed at least 1 week prior to the first experimental session. The 2 experimental sessions were completed 1 week apart for 20 of the 27 participants. The experimental sessions for the other participants were scheduled between 6 and 17 days apart owing to scheduling constraints. All sessions started in the morning or early afternoon (1000–1330 h) appropriately 3 h after breakfast ingestion. For a given participant the start time of the second session was always within 30 min of the first session. During each session, participants completed an 11-min BWE protocol or an equivalent period of sitting that constituted the CON condition.

### Pre-experimental procedures

Participants completed a short medical questionnaire as part of the initial screening and intake process. The questions related to their health status, medication use, allergies, dietary preferences, or restrictions, and, for the female participants, information regarding any contraceptive use and their menstrual cycle. Female participants who were currently using some form of contraception (e.g., pill, IUD, ring; n = 8) completed both experimental trials during the active hormone period rather than the placebo phase. Naturally cycling female participants (n = 11) completed both experimental trials between day 1–15 of their cycles which typically corresponds to the follicular phase^12^. For the second study visit, participants watched a short video in our laboratory to inform them about the CGM device (Abbott Libre Sense Glucose Sport Biosensor) and insertion procedures. Height and body mass were also measured during this visit prior to the completion of a graded exercise test to volitional fatigue on a cycle ergometer (Lode Excalibur Sport version 2.0, Groningen, The Netherlands). Peak power output was recorded at the end of this test to calculate maximal oxygen uptake (VO_2max_) using a validated equation as previously described^6,13^. The fitness test consisted of a 4-min warm-up at 0 watts (W) followed by a stepwise increase in intensity of 15 W per minute thereafter. Participants were asked to maintain a cadence between 70 and 90 revolutions per minute (rpm) during the test and the test was stopped when cadence fell below 60 rpm, at which point peak power output was recorded. Participants were given a study kit for use at home during the virtual sessions which included a Polar HR monitor (Polar H7 Bluetooth Smart Heart Rate Sensor & Strap, Kempele, Finland), an activity monitor (ActiGraph wGT3X-BT v1.9.2, Pensacola, FL, USA), and a 6–20 Borg rating of perceived exertion (RPE) scale^14^. Participants subsequently completed a virtual exercise familiarization which involved performing the 11-min BWE protocol remotely under the supervision of a researcher over a video call. This enabled the participants to become accustomed to the protocol before the main experimental trials. HR was measured during the exercise protocol and RPE was obtained immediately upon completion of the last exercise bout. Participants were given the option to subsequently insert the CGM device on the back of their upper arm ~ 24 h prior to the first experimental trial or have a study investigator perform the insertion for them. A researcher delivered individualized frozen, pre-packaged meals prepared by a commercial company (Heart to Home Meals) and 2 glucose drinks (75 g each) (NERL™ Trutol™ Glucose Tolerance Test Beverages, Thermo Fisher Scientific, Waltham, MA, USA; Unflavored OGTT, VWR Scientific, Radnor, PA, USA) to the participants before the experimental sessions.

### Experimental trials

Each trial involved a controlled nutritional period that commenced in the morning before the 11-min BWE or CON protocol and concluded 24 h after each protocol. Participants were instructed to only consume the food that was provided to them during this period. In the event a participant consumed an additional snack during the first trial, they were asked to replicate this during the second trial for consistency. The macronutrient breakdown of each meal was ~ 50% carbohydrates, ~ 30% fats, and ~ 20% protein. Daily total caloric need of each participant was calculated using the Harris–Benedict equation with the activity factor set to 1.4^15,16^. Based on these calculations, the mean energy intake over 24 h was 2260 ± 380 kcal which included the energy supplied from breakfast, lunch, and dinner. The first controlled meal (breakfast the morning before the remote experimental trial) made up ~ 25% of the daily energy total in kcal. The second, third, and fourth meals (lunch, dinner, and breakfast the next morning) made up ~ 30%, ~ 45%, and ~ 25% of the total daily energy, respectively. Participants were instructed to consume the same individualized meals at the same times during both experimental trials. Participants were allowed to consume any beverages they wanted, except those containing alcohol, but they were asked to replicate beverage consumption patterns between the experimental trials. Participants were provided with a meal timing and beverage log to facilitate the replication process. All participants were asked to consume their first controlled meal in the morning 3 h prior to the start of the BWE or CON protocol. Participants completed the 11-min BWE or CON protocol remotely under the supervision of a researcher on video call. Participants remained on the video call with a researcher for 1 h after completing each 11-min protocol. After the rest period, participants were given 5 min to ingest the 75 g oral glucose drink under the supervision of the researcher on the video call.

Glycemic parameters were assessed by CGM during the 24 h period immediately following the 11-min BWE or CON condition. Participants were asked to eat the second meal (lunch) at least 2 h after consuming the 75 g glucose drink and the third meal (dinner) at least 2 h after lunch. This was scheduled to allow a distinct 2 h postprandial window to assess glycemic responses to the 75 g glucose drink and each controlled meal. Participants were instructed to refrain from structured exercise for the 24 h period while glycemic responses were measured. Activity data, including total energy expenditure (kcal), mean metabolic equivalents (METs), mean sedentary time (percent), and total steps, was also collected over this time period using a wrist-worn activity monitor (ActiGraph). The ActiLife 6 Data Analysis Software (v6.13.4, ActiGraph, Pensacola, FL, USA) compelled the recorded activity data into csv files such that these specific activity outcomes could be calculated within Excel. HR was measured to characterize the intensity of the 11-min BWE protocol vs the CON protocol. Polar HR chest straps were worn during each protocol, and they were paired via Bluetooth to the ActiGraph wrist-worn devices to record HR data. The HR data for each 11-min protocol was extrapolated from the ActiLife 6 Data Analysis Software into csv files where the mean HR and peak HR were calculated within Excel. After the completion of both experimental trials, participants returned to the laboratory to have the CGM device removed and to return the related study materials within the study kit.

### BWE protocol

The 11-min BWE protocol was modelled on one used in a previous study in our laboratory^17^. The specific exercises were modified to reduce joint impact forces with the goal of increasing the accessibility and translatability of the protocol. The protocol started with 60 s of jumping jacks to warm-up and was followed by 5 exercises performed for 60 s each and interspersed with 60 s periods of walking on the spot for recovery. The protocol ended with 60 s of walking on the spot to cool-down. The specific exercises that were performed in order were: squat thrusts (modified burpees); knee tucks (left leg for 30 s, right leg for 30 s); mountain climbers; knee tucks (left leg for 30 s, right leg for 30 s); and squat thrusts again. The BWE protocol was facilitated by having participants follow along to a custom workout video made by a researcher that demonstrated the entire exercise protocol. Participants were encouraged to complete as many repetitions as possible for each of the 5 exercises in the allotted time.

### Determination of glycemic outcomes

The raw CGM data was automatically uploaded to an online cloud-based system (Supersapiens Dashboard, ATL, USA). Data files containing interstitial glucose data in 1 to 15-min increments were subsequently downloaded for each participant. Glycemic parameters were subsequently determined based on the 24 h period that started with the minute following completion of BWE or CON protocol. An open access Excel spreadsheet (EasyGV 9.0.R2, University of Oxford, England, United Kingdom) as previously described^15^ was used to calculate the 24 h mean glucose as well as MAGE over the 24 h. Glucose CV, which is a marker that provides insight into glucose “stability”^18^, was calculated as follows: SD/24 h mean glucose. Postprandial 2 h glucose means, postprandial glucose peaks, and meal excursions were calculated within Excel. Postprandial periods included the 2 h window after the consumption of the 75 g glucose drink, lunch, dinner, and breakfast the following morning. The 2 h postprandial glucose means were calculated as the average glucose over each respective 2 h window. Postprandial glucose peaks were recorded as the maximum glucose concentrations achieved in each respective 2 h window. Meal excursions were calculated by subtracting the pre-meal baseline glucose from the postprandial peak glucose concentration. The pre-meal baseline glucose was determined to be the 15-min mean glucose prior to the meal start time. The CGM devices utilized in this study have a standard glucose reading interval of < 1–15 min under normal measurement conditions. Missing data was identified as any glucose reading interval of > 15.5 min. The percentage of missing data over the 24 h measurement period was calculated by determining the number of minutes missing above and beyond the largest glucose sampling interval (15 min in this case) and then dividing the total number of missing minutes by the number of minutes in 24 h (1440 min). The percentage of missing data over a 2 h postprandial period was calculated in the same way except, the total number of missing minutes in the respective 2 h window was divided by 120 min instead. We set 10% as the missing data cut-off threshold, such that participants with more than 10% missing CGM data over the entire 24 h period or in any 1 of their 2 h postprandial periods would be excluded from the respective analysis. In our data set, n = 9 participants had no missing data and n = 18 had small portions of missing data throughout the 24 h measurement periods. Among these participants the approximate average percentage of missing data across the 24 h period was < 2%. Therefore, no participants exceeded the cut-off threshold for the entire 24 h period, however, 2 participants exceeded the missing data threshold for the postprandial breakfast response during 1 experimental session. These 2 participants were excluded from the breakfast postprandial analysis (postprandial glucose peak, 2 h postprandial mean glucose, and meal excursion) and therefore, those outcomes are based on n = 25. The day-to-day variability of the CGM measurements was determined by comparing the postprandial glycemic responses to the first controlled meal consumed 3 h prior to the start of each experimental trial. The 2 h postprandial glucose means, postprandial glucose peaks, and meal excursions were compared between the 2 different days to determine the level of variability across days within the same participant. Technical error (TE) was calculated according to the following equation^19^:

$$ {\text{TE }} = \, (SDdiff \div \, \surd {2} \div grand\;mean) \, \times { 1}00. $$

### Statistical analysis

The independent variable was condition (BWE vs CON), and the dependent variables were 24 h mean glucose, 2 h postprandial mean glucose, postprandial glucose peaks, meal excursions, glycemic variability, and activity factors including total kcals, total steps, average METs, and average percent in sedentary behaviour. All data that were normally distributed were analyzed using a one-tailed paired t-test. A Shapiro–Wilk test was performed to assess normality of the data set. If the data set was not normally distributed, a Wilcoxon signed-rank test was used which is the non-parametric equivalent^20^. For glycemic outcomes listed above, outliers were identified by creating a modified boxplot for the data set and implementing the use of quartiles and the interquartile range^21^. If a data point sat below the first quartile or above the third quartile by more than 1.5 times the interquartile range, then the data point was deemed an outlier in the data set^21^. There were no outliers detected for the postprandial peak glucose or meal excursion following the 75 g glucose drink as well as CV. The remaining CGM outcome measures had between 1 and 4 detectable outliers depending on the specific outcome of interest. Statistical analysis of the CGM data was performed with and without inclusion of outliers; this did not change the interpretation of the result in any case. Significance for all analyses was set as p ≤ 0.05. Analyses were performed using GraphPad Prism 9.3.1 (GraphPad Software Inc., California, USA). All data are presented as mean ± SD for n = 27 except where noted.

## Results

### Descriptive data

**Figure 3:**
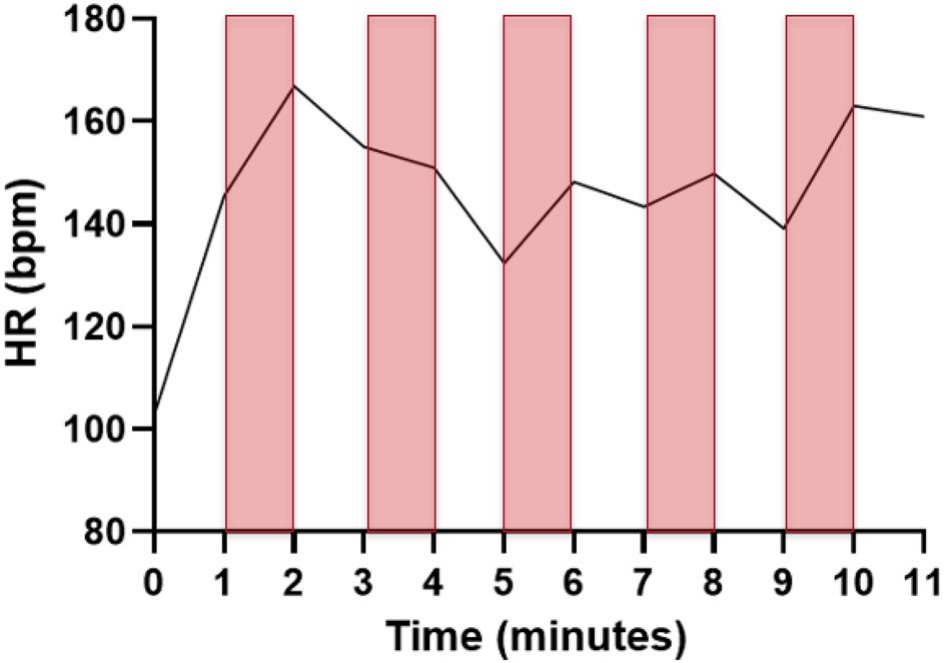
Representative heart rate tracing for 1 participant during the 11-min bodyweight exercise (BWE) protocol. The bars correspond to the 5, 1-min BWE intervals.

Peak power achieved during the progressive cycling fitness test to volitional fatigue was 198 ± 63 W. VO_2max_ calculated from the fitness test was 36 ± 7 mL/kg/min. RPE for the BWE protocol was 14 ± 2 vs 6 ± 0 for CON. Peak HR elicited during the BWE protocol was 173 ± 14 bpm and the mean HR was 147 ± 14 bpm (n = 26). These values corresponded to 88% and 75%, respectively, of age-predicted maximal HR. The mean HR for the CON was 73 ± 11 bpm (n = 22). Figure 3 illustrates a representative HR tracing for 1 participant during the 11-min BWE protocol.

### Activity data

Total kcals, total steps, average METs, and average percent time in sedentary behaviour are summarized in Table 2. There were no differences between conditions for any variable (p > 0.05).

**Table 2:** Activity monitoring data.

Activity measure	BWE	CON	p-value
Total kcals	1222 ± 771	1084 ± 536	0.27
Total steps	10,983 ± 5134	10,152 ± 3725	0.20
Average METs	1.40 ± 0.24	1.34 ± 0.16	0.15
Average percent sedentary (%)	54 ± 9	54 ± 12	0.46

p-values are based on a Wilcoxon matched-pairs signed rank test for all outcomes except average percent sedentary time which was assessed using a paired t-test (n = 25).

*BWE* bodyweight exercise, *CON* control, sitting, *METs* metabolic equivalents.

### CGM data

**Figure 4:**
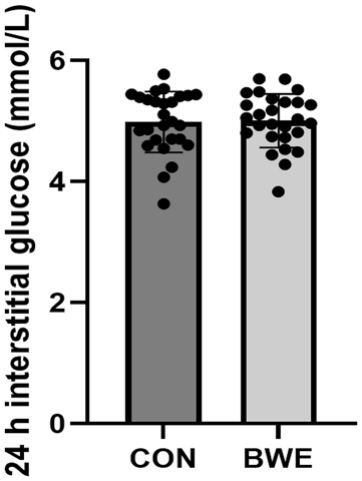
24 h mean glucose concentrations following bodyweight exercise (BWE) or control condition (CON).

**Figure 5:**
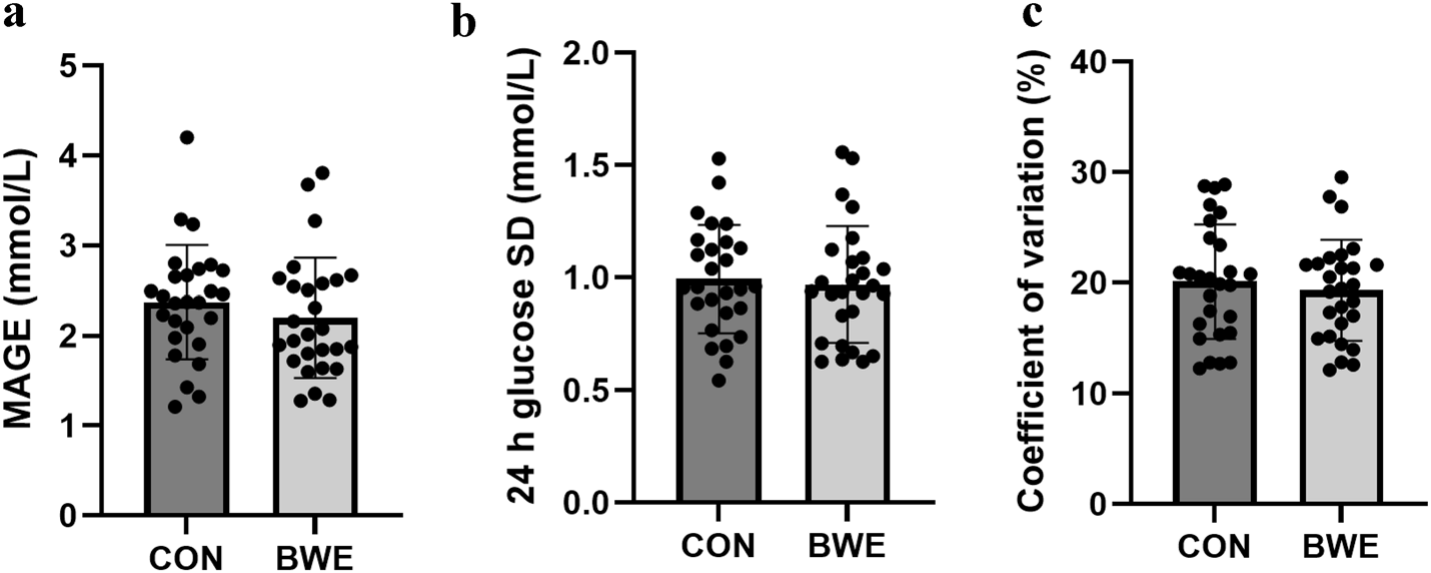
Measures of 24 h glycemic variability (p > 0.05). Mean amplitude of glycemic excursions (MAGE) (**A**), standard deviation (SD) (**B**), and coefficient of variation (CV) (**C**). *BWE* bodyweight exercise, *CON* control, sitting.

**Figure 6:**
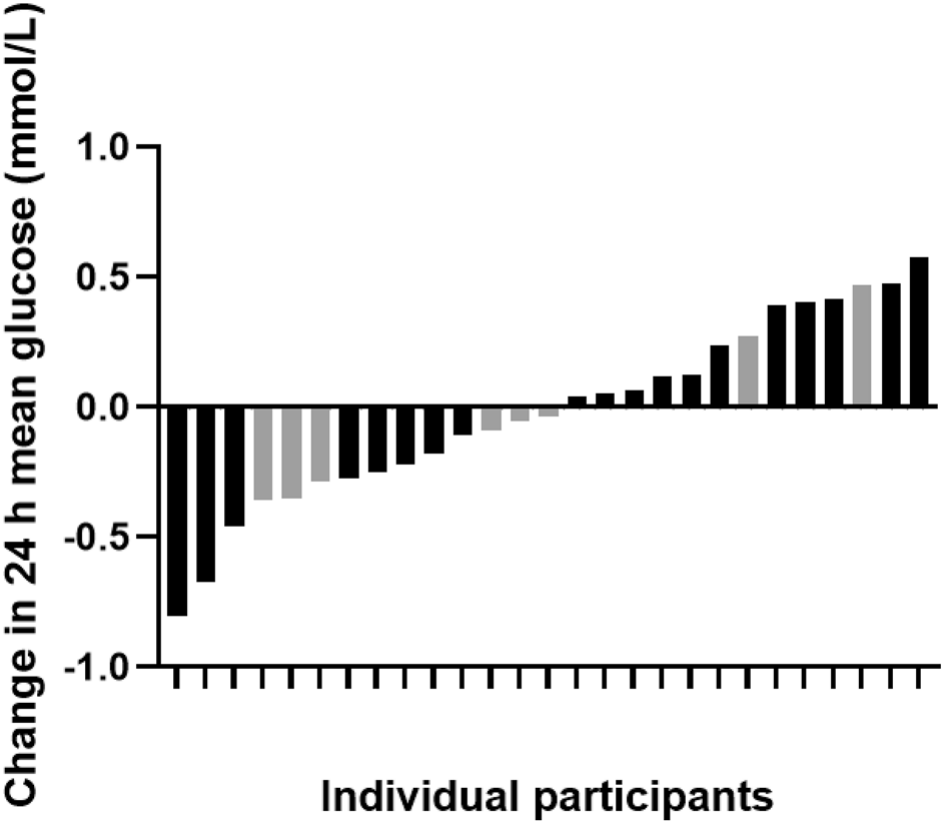
Individual responses of the change in 24 h mean glucose between the CON and BWE conditions. Each bar represents data for 1 participant where the change score = CON value minus BWE value. Positive net values represent participants whose 24 h mean glucose was greater in the CON condition whereas negative values coincide with the 24 h mean glucose being greater in the BWE condition. Gray bars represent male participants and darker bars represent female participants.

The 24 h mean interstitial glucose concentration was not different between BWE vs CON (5.0 ± 0.4 mM vs 5.0 ± 0.5 mM, p = 0.39; *d*_*z*_ = 0.06; Fig. 4). The 95% confidence interval (CI) for 24 h mean glucose was 4.8–5.2 mM for both BWE and CON. There were no differences between BWE and CON for any measure of glycemic variability including MAGE, SD, and CV in the 24 h following the interventions (p > 0.05; Table 3, Fig. 5). There was no difference in any postprandial glucose measure after BWE vs CON (p > 0.05; Table 4). There were also no differences in the postprandial glucose measures following the ingestion of an identical breakfast 3 h prior to the start of each experimental trial on 2 different days (p > 0.05; Table 5). The mean baseline pre-meal glucose value before the consumption of the first controlled breakfast on trial day 1 was 4.8 ± 0.6 mM versus 4.8 ± 0.5 mM on trial day 2. Additionally, there were no differences in the 1 h post-intervention glucose means between BWE vs CON (p > 0.05). The individual participant responses of the change in 24 h mean glucose between the BWE and CON conditions are summarized in Fig. 6.

**Table 3:** Measures of glycemic variability.

Glycemic variability measure	BWE	BWE 95% CI	CON	CON 95% CI	Mean difference between conditions	p-value	d_z_
MAGE (mM)	2.2 ± 0.7	1.9–2.5	2.4 ± 0.6	2.1–2.6	0.17	0.06	− 0.31
SD (mM)	1.0 ± 0.3	0.9–1.1	1.0 ± 0.2	0.9–1.1	0.02	0.30	− 0.10
CV (%)	19 ± 5	18–21	20 ± 5	18–22	1	0.21	− 0.16

*BWE* bodyweight exercise, *CI* confidence interval, *CON* control, sitting, *CV* coefficient of variation, *d*_*z*_ effect size, *MAGE* mean amplitude of glycemic excursions, *mM* mmol/L, *SD* standard deviation.

p-values are on a paired t-test for all outcomes of glycemic variability.

**Table 4:** Postprandial glucose responses.

A—Postprandial period	BWE peak (mM)	BWE 95% CI (mM)	CON peak (mM)	CON 95% CI (mM)	p-value	*d*_*z*_
Glucose drink	8.8 ± 1.3	8.3–9.4	8.9 ± 1.3	8.4–9.5	0.35	− 0.07
Lunch	7.0 ± 1.4	6.5–7.6	6.9 ± 1.1	6.4–7.3	0.35	0.08
Dinner	7.3 ± 1.5	6.7–7.9	7.0 ± 1.1	6.5–7.4	0.11	0.25
Breakfast	6.5 ± 1.1	6.1–7.0	6.7 ± 1.4	6.1–7.3	0.26	− 0.13

B—Postprandial period	BWE 2 h mean (mM)	BWE 95% CI (mM)	CON 2 h mean (mM)	CON 95% CI (mM)	p-value	*d*_*z*_
Glucose drink	6.6 ± 0.8	6.3–6.9	6.7 ± 0.9	6.3–7.0	0.34	− 0.08
Lunch	*5.2* ± *0.9*	*4.9–5.6*	*5.1* ± *0.6*	*4.8–5.3*	*0.31*	*0.19*
Dinner	*5.6* ± *1.0*	*5.2–6.0*	*5.4* ± *0.6*	*5.1–5.6*	*0.23*	*0.23*
Breakfast	4.9 ± 0.5	4.7–5.1	4.9 ± 0.7	4.7–5.2	0.43	− 0.04

C—Postprandial period	BWE meal excursion (mM)	BWE 95% CI (mM)	CON meal excursion (mM)	CON 95% CI (mM)	p-value	*d*_*z*_
Glucose drink	4.0 ± 1.5	3.4–4.6	4.2 ± 1.6	3.6–4.8	0.19	− 0.17
Lunch	1.9 ± 1.6	1.3–2.6	1.7 ± 1.7	1.0–2.4	0.30	0.10
Dinner	*2.3* ± *1.3*	*1.8–2.9*	*1.9* ± *1.4*	*1.4–2.5*	*0.12*	*0.28*
Breakfast	1.9 ± 0.8	1.6–2.3	2.0 ± 1.0	1.6–2.4	0.35	− 0.08

*BWE* bodyweight exercise, *CI* confidence interval, *CON* control, sitting, *d*_*z*_ effect size, *mM* mmol/L.

*BWE* bodyweight exercise, *CI* confidence interval, *CON* control, sitting, *d*_*z*_ effect size, *mM* mmol/L.

Italicized numbers denote analyses based on a Wilcoxon signed-rank test because the data did not pass the normality test. Postprandial glucose peaks (Table A), 2 h postprandial mean glucose (Table B), and meal excursions (Table C).

**Table 5:** Day-to-day variation in CGM indices determined after a standardized breakfast.

	Glucose peak (mM)	2 h mean (mM)	Meal excursion (mM)
Day 1	7.0 ± 1.3	5.2 ± 0.7	*2.1* ± *1.0*
Day 2	6.9 ± 1.1	5.1 ± 0.5	*2.1* ± *1.0*
p-value	0.27	0.22	*0.44*
TE (%)	11.9	7.5	37.3

Postprandial glycemic responses after ingestion of a standardized breakfast before the start of the experimental trials.

*mM* mmol/L, *TE* technical error.

Italicized values denote analyses based on a Wilcoxon signed-rank test.

## Discussion

The main finding from this study was that an 11-min BWE protocol did not alter 24 h glycemic responses determined by CGM in young, healthy, inactive adults. In contrast to our hypothesis, we found no differences in 24 h mean glucose or indices of glycemic variability or postprandial glycemic responses after the BWE session as compared to a non-exercise CON condition. It is unclear whether the lack of effect was because the exercise stimulus was insufficient to alter glycemic responses in our cohort of relatively young healthy participants. It is possible that the glucose control of these individuals was already robust with relatively limited potential to be enhanced by an acute session of exercise like that used in our study. It is also possible that the timing of exercise relative to the meals or glucose drink ingestion may have influenced the results. This study nonetheless demonstrates the feasibility of performing BWE remotely while simultaneously utilizing CGM in a free-living situation.

Little et al*.*^6^ previously reported no difference in 24 h mean glucose measured with CGM after participants completed a vigorous intermittent exercise session on a cycle ergometer (10, 60-s bouts at an intensity corresponding to ~ 90% of peak HR) compared to a control condition. Similarly, the present study found no change in 24 h mean glucose following exercise compared to the CON condition. In contrast Little et al*.*^6^, found improvements in some measures of postprandial glycemic control whereas we found no differences in any postprandial glycemic responses. This may be due to differences in the study cohort, the number and intensity of exercise intervals, or the different modality of exercise used between the studies. Little et al*.*^6^ recruited participants who were on average 18 years older and had a BMI that was 11 kg/m^2^ greater than the BMI of our participants. It is possible that the group of younger, adults with lower BMI in the present study were less responsive to a short bout of intermittent exercise as compared to older adults who were living with overweight or obesity. Additionally, more than 50% of the participants in the study by Little et al.^6^ had impaired fasting glucose at baseline. Individuals with impaired glucose control may be more responsive to an acute bout of brief, intermittent exercise as compared to our participants who self-reported having no previous diagnosis of a metabolic health condition. Furthermore, our study involved 5 × 60 s BWE intervals over an 11-min period whereas Little et al*.*^6^ had participants perform 10 bouts of high-intensity cycling intervals over a ~ 20 min session. Whereas the mean intensity elicited during the BWE bouts in the present study can be characterized as ‘vigorous’, based on common methods in authoritative guidelines for exercise testing and prescription (i.e., RPE and percentage of age-predicted maximal heart rate)^22^, the mean intensity over the entire session was lower than in the study by Little et al.^6^ It is also possible that a larger ‘dose’ or total volume of BWE (i.e., a greater number of intervals and/or intervals performed at a higher relative intensity) may be needed to elicit responses in relatively young, healthy individuals. We do not feel that the virtual nature of the intervention per se was the reason for the lack of response, as previous studies have shown that virtually-monitored, home-based BWE interventions can elicit training responses like traditional lab-based high-intensity interval training interventions^23^.

Two previous studies^7,8^ also showed an effect of BWE on postprandial glycemic control over a 1–2 h period postexercise. Barillas et al*.*^7^ measured glycemic responses using capillary blood samples at specific time points during the 1 h after consuming a glucose drink whereas Solomon et al.^8^ measured glycemic indices over 2 h using CGM. Solomon et al*.*^8^ only saw a positive effect when breakfast was consumed immediately before exercise, whereas no effect was seen when breakfast was consumed 30 min before exercise or immediately after completing exercise. This suggests that the time course between nutritional manipulations and exercise can influence postprandial glycemic responses. The timing of our nutritional manipulations surrounding exercise differed from these previous studies and this may in part explain the differences observed in postprandial glycemic responses. Solomon et al.^8^ utilized CGM, but they only reported a 2 h window of data. Our study expands upon the current literature by introducing the use of CGM to measure 24 h glycemic responses to acute BWE which, to our knowledge, has not previously been done. Differences in the results of the present study compared to the study by Barillas et al*.*^7^ could be attributed to differences in the study cohorts as well. Barillas et al.^7^ recruited participants who had previously engaged in resistance and plyometric exercise on a regular basis leading up to their participation. Additionally, it was a requirement that participants could attain 80% of their age predicted HR maximum when completing plyometric exercise^7^. Since these participants were experienced with plyometrics, it is possible they exerted a higher level of effort during the workout reflected by a higher HR response. It has been suggested that a relative intensity of ≥ 80% of HR maximum may be the intensity threshold needed to see changes in glycemic responses postexercise^7^. As previously noted, our participants achieved a mean HR equivalent to 75% of maximum over the 11-min bout. Our participants performed the BWE protocol at a self-selected pace to mimic at-home BWE workouts where individuals may not have the necessary equipment to monitor their HR or the intensity of the exercises.

The lack of change in glycemic control postexercise may also be related to the nutritional state of participants. Previous research^24^ has indicated that fasted state exercise may promote beneficial changes in glycemic control. Terada et al.^24^ found that completing an acute bout of treadmill exercise in the fasted state was more advantageous for postprandial glycemic control and glycemic variability (MAGE) compared to exercising in a fed state in people with type 2 diabetes. In the present study, we had participants consume breakfast 3 h prior to exercise. The glycemic control responses may have differed if the BWE was completed in fasted state instead. It is also possible that exercise nutritional state may have a greater impact on those with impaired glycemic control including individuals with type 2 diabetes as compared to our study cohort of young, healthy adults. The glycemic index of the controlled meals provided to the participants in our study could have influenced the postprandial glucose responses. Each participant consumed the same meals in both trials therefore, the glycemic index of the meals was consistent within each participant, however all participants had different individualized meal plans. Hence, the glycemic index of the meals was not standardized across participants. Campbell et al.^25^ demonstrated that glucose area under the curve was larger after consuming high glycemic index foods compared to low glycemic index foods in individuals with type 1 diabetes. This suggests the possibility of greater glucose responses in some participants compared to others depending on the glycemic index of the foods in their meal plans. The present study opted for an individualized meal plan approach which was based on the height, body mass, age, and sex of each participant. If standard meal plans were provided to all participants whereby everyone consumed identical meals with the same nutritional profile and glycemic index then each participant would have been in a different nutritional state (caloric deficit, maintenance, or surplus) because height, body mass, age, and sex would not have been accounted for.

From a mechanistic standpoint, vigorous exercise elicits a variety of physiological responses that could influence indices of glycemic control postexercise. During an exercise session and for ~ 2 h postexercise, glucose supply to the skeletal muscle cells is elevated and there is also an enhanced capacity to uptake glucose into contracting skeletal muscle^26^. A greater level of glucose uptake postexercise would lead to lower blood glucose concentrations and this would be interpreted as an improvement in glycemic control. However, simultaneously there are competing mechanisms which help to maintain, or even increase, blood glucose concentrations. For example, intense exercise can elicit increases in catecholamine concentrations which promotes an increase in hepatic glucose production^27,28^ that can increase glucose concentrations by as much as ~ 5.0 mM^9^. It is evident that there are both positive and negative signals at work in the postexercise period which might lead to no change in glycemic control observed if these signals are in a relative equilibrium. It is possible that the BWE protocol in our study elicited a number of competing mechanisms which ultimately resulted in no change observed in glycemic control compared to the CON condition. There was also a wide range of responses to BWE vs CON where 13 participants experienced a lower 24 h mean glucose following BWE compared to CON, and 14 participants experienced a higher 24 h mean glucose following BWE compared to CON (Fig. 6). This observation highlights the individual variation in 24 h glycemic responses following BWE. We also found the postprandial glycemic responses following the consumption of the same meal on 2 different days ~ 1 week apart were not different. This suggests that the CGM device employed for the glycemic measurements in this study can reproduce the glucose measurements reasonably well day to day.

Future studies are needed to further explore the effects of BWE on glycemic control in healthy individuals and in individuals with impaired glucose control. For example, studies exploring larger doses of acute BWE to help determine the minimum exercise prescription required to see an effect in this population would be warranted. Additionally, investigating the effects of repeated bouts of BWE in the form of training studies would be useful to explore the potential for chronic exercise to alter glycemic control. Also, studying the effects of various nutritional manipulations surrounding BWE interventions would be helpful. For example, investigating how glycemic control is influenced by fasted vs fed state exercise, the timing of meals surrounding exercise, as well as the glycemic indices of foods consumed. Our study was powered to detect changes in our primary outcome of 24 h mean glucose, therefore future adequately powered studies should further probe the impact of BWE on glycemic variability given the fact that our MAGE data trended towards showing a benefit from BWE although this was not statistically significant (p = 0.06). Additionally, future work should explore whether there are sex-based differences in glycemic responses to BWE. Also, studies investigating the mechanistic basis behind observed increases, decreases, or no changes seen in glycemic control following exercise would be warranted. Further, including measures of counter-regulatory hormone responses (i.e., insulin action) in future work would be valuable since it is possible that acute exercise protocols could alter insulin concentrations without an observed change in glucose. Such studies should be conducted in a variety of study populations such as healthy adults, individuals with impaired glucose control, or those at risk of developing metabolic diseases. Perhaps other study populations would be more sensitive to the effects of BWE on glycemic control compared to the young, healthy adults we recruited.

Some strengths of the present study included the level of nutritional control, the use of CGM in a free-living situation, the recruitment of both sexes, as well as the menstrual cycle control implemented with the female participants. Whereas some limitations of the design included the use of self-reported dietary logs, conducting remote visits which created less environmental or laboratory-based control, as well as the lack of counterbalancing the number of males and females. We provided participants with controlled, pre-packaged meals to consume during the 24 h CGM measurement period. This was a strength of the study design because it allowed for an enhanced level of nutritional control within each participant, ensuring that they consumed the same meals on both trials. Meal and beverage consumption patterns (i.e., timing and amount consumed) were self-reported by the participants in a meal and beverage log. This could be considered a limitation of the study given the potential difficulties surrounding self-reported data. We utilized CGM which is considered a very detailed and sensitive measurement tool that produces comprehensive glucose data. This study was designed to simulate a practical, free-living situation where the intervention was completed remotely at-home and the use of CGM facilitated this design due to the fact that participants did not have to be in the lab for the collection of glucose data. Although conducting a free-living study comes with its own benefits it also removes aspects of lab-based studies which have a superior level of environmental and activity control. For example, the free-living nature of this study resulted in the activity levels of our participants not being standardized in the 2 h following the consumption of the 75 g glucose drink which could be considered a limitation. A strength of our study was the unbiased recruitment of both males and females. We also controlled for menstrual cycle in our female participants to ensure that hormonal profiles were similar during both trials. However, we did not counterbalance for sex in our recruitment and therefore, we ended up with more females than males (n = 19 vs n = 8 respectively). This limitation may be particularly relevant since Gillen et al*.*^29^ showed that 6 weeks of sprint interval training improved 24 h mean glucose measured with CGM in males but not in females. This suggests that females could have a blunted glycemic response to exercise interventions which should be taken into consideration given that 70% of the participants in the present study were females.

## Conclusion

This study found that an 11-min BWE protocol did not alter glycemic responses as compared to an equivalent seated control period, during the subsequent 24 h under conditions of standardized nutritional intake. There were no differences between conditions for 24 h mean glucose, measures of glycemic variability, and the postprandial glycemic outcomes. The BWE protocol employed did not provide a sufficient stimulus to alter glycemic responses in this cohort of young, healthy adults. Future studies should investigate the influence of BWE in people with impaired glycemic control as well as potential changes over time in response to a BWE training intervention. The relationship between BWE and 24 h MAGE also warrants further investigation as our study may have been underpowered to detect this specific relationship.

## Data Availability

Data can be made available upon request to the corresponding author.
